# Descriptive inference using large, unrepresentative nonprobability samples: An introduction for ecologists

**DOI:** 10.1002/ecy.4214

**Published:** 2024-01-12

**Authors:** Robin J. Boyd, Gavin B. Stewart, Oliver L. Pescott

**Affiliations:** ^1^ UK Centre for Ecology & Hydrology Wallingford UK; ^2^ Evidence Synthesis Lab School of Natural and Environmental Science, University of Newcastle Newcastle‐upon‐Tyne UK

**Keywords:** bias, biodiversity monitoring, nonprobability samples, weighting

## Abstract

Biodiversity monitoring usually involves drawing inferences about some variable of interest across a defined landscape from observations made at a sample of locations within that landscape. If the variable of interest differs between sampled and nonsampled locations, and no mitigating action is taken, then the sample is unrepresentative and inferences drawn from it will be biased. It is possible to adjust unrepresentative samples so that they more closely resemble the wider landscape in terms of “auxiliary variables.” A good auxiliary variable is a common cause of sample inclusion and the variable of interest, and if it explains an appreciable portion of the variance in both, then inferences drawn from the adjusted sample will be closer to the truth. We applied six types of survey sample adjustment—subsampling, quasirandomization, poststratification, superpopulation modeling, a “doubly robust” procedure, and multilevel regression and poststratification—to a simple two‐part biodiversity monitoring problem. The first part was to estimate the mean occupancy of the plant *Calluna vulgaris* in Great Britain in two time periods (1987–1999 and 2010–2019); the second was to estimate the difference between the two (i.e., the trend). We estimated the means and trend using large, but (originally) unrepresentative, samples from a citizen science dataset. Compared with the unadjusted estimates, the means and trends estimated using most adjustment methods were more accurate, although standard uncertainty intervals generally did not cover the true values. Completely unbiased inference is not possible from an unrepresentative sample without knowing and having data on all relevant auxiliary variables. Adjustments can reduce the bias if auxiliary variables are available and selected carefully, but the potential for residual bias should be acknowledged and reported.

## INTRODUCTION

As the data revolution gathers pace, it is not surprising to see “big data” being used to monitor biodiversity. Examples include observations submitted to mobile phone apps by amateur naturalists (Johnston et al., 2022) and digitized specimens from museums and herbaria (Nelson & Ellis, 2019). Such data become bigger still when combined in data aggregators such as the Global Biodiversity Information Facility (GBIF; https://www.gbif.org/) or metadatabases such as PREDICTS (Hudson et al., 2014). Unfortunately, quantity of data does not necessarily imply quality of insight.

Monitoring biodiversity is typically a matter of descriptive statistical inference. It is inferential in that the goal is to infer something about a target population from a sample of that population (Boyd, Powney, et al., 2023). The population might comprise, say, all areal units across some landscape (“sites”), in which case the sample would be a subset of those sites. The inference is descriptive in that the aim is to describe (rather than explain) a variable of interest in the population. A common example is the proportion of sites occupied by some species (Bowler et al., 2021; Outhwaite et al., 2020; Powney et al., 2019; Stroh et al., 2023; van Strien & van Grunsven, 2023), but there are many others.

Of more importance than the size of a sample for descriptive inference is whether it is representative of the population about which inferences are to be drawn (Meng, 2018). In a representative sample, the distribution of the variable of interest is similar to its distribution in the population (Bethlehem et al., 2008). An equivalent definition is that there is little to no correlation between inclusion in the sample and the variable of interest, the “data defect correlation” or ddc (Meng, 2018). Intuitively, statistics derived from a representative sample, such as means and proportions, will be similar to their population equivalents.

Unfortunately, ddcs are likely to be appreciable in big biodiversity datasets. For one, naturalists preferentially visit and collect data at sites where they are likely to see species that interest them (Bowler et al., 2022; Forister et al., 2023). When those species' abundances or distributions are the variables of analytic interest, preferential sampling naturally results in a positive ddc (McClure & Rolek, 2023). On the other hand, naturalists might be constrained to visiting and collecting data in, say, built‐up areas, which are easier to access than remote locations (Geldmann et al., 2016; Hughes et al., 2020; Mandeville et al., 2022). Built‐up areas generally have low‐quality habitats, meaning that species are less likely to occupy them in large numbers and that the ddc might be negative.

Inferences from unrepresentative samples, with appreciable ddcs, are likely to be misleading. Imagine a researcher who wants to estimate the average abundance of some species across a landscape. An obvious (but naive) approach would be to calculate its mean abundance across sampled sites and assume that this is similar to its average abundance across the wider landscape. However, if the locations at which the species is most abundant were preferentially sampled, then the sample‐based estimate of its mean abundance will be upwardly biased. To use the analogy of Forister et al. (2023), sampled locations would be life rafts; nonsampled locations would be the sinking ship.

It is simple to counteract the biasing effect of the ddc if the probability that each site was included in the sample is known; that is if a probability sample is available. In this case, more weight can be placed on the data from sites that were less likely to be included. The effect of this type of weighting is easiest to explain heuristically: the sample is augmented with “copies” of the data from sites that were less likely to be sampled, effectively bringing sample inclusion probabilities across sites to parity. Two variables cannot be correlated if one of them is constant, which means there can be no correlation between the weighted sample inclusion probabilities and the variable of interest across sites. It follows that the ddc, which is the correlation between actual (weighted) sample inclusion and the variable of interest, is zero in expectation (Meng, 2022), and the sample can be considered representative (Lohr, 2022). Weighting of this type is known as “design‐based” inference, because the inclusion probabilities are a feature of the sampling design.

Design‐based inference is not applicable for the types of big biodiversity datasets we consider here, because they were not collected according to a probabilistic sampling design. We do not know the probability that sites were visited by the collectors of specimens now held in museums and on GBIF. Nor do we know the probability that citizen scientists visited and collected data at each site across most landscapes. Matters are simpler when using data from structured monitoring schemes, which often aim for a probability sample (e.g., the UK Pollinator Monitoring Scheme; UK PoMS, 2023). However, the incomplete uptake of sites that were selected for inclusion (Pescott et al., 2015, 2019) means that, in practice, these samples too are nonprobabilistic. (Incomplete uptake in biodiversity monitoring is analogous to the issue of nonresponse in survey sampling, e.g., Bethlehem et al., 2008.) When sample inclusion probabilities are not known, an alternative to design‐based inference is needed.

Most approaches to inference from nonprobability samples involve estimating the inclusion probabilities. A relatively simple example is poststratification, where the observations (for each site) are split into strata based on covariates, and sites in strata that are underrepresented in the population (based on the population totals of the covariates) are given more weight (Valliant et al., 2018). Using covariates to estimate sample inclusion probabilities is equivalent to adjusting the samples in such a way that the distributions of those covariates in the sample more closely resemble their distributions in the population (i.e., across all sites in the wider landscape). If the covariates affect both the variable of interest and sample inclusion, then inferences drawn from the adjusted sample will be closer to the truth than those from the original (naive) sample. In the context of inference from nonprobability samples, covariates affecting both sample inclusion and the variable of interest, which are not of direct analytic interest themselves, are known as “auxiliary variables” (Thoemmes & Mohan, 2015; Thoemmes & Rose, 2014).

Before going further, it is important to note that most approaches to inference from nonprobability samples rest on the bold assumption that the variable of interest is independent of sample inclusion after accounting for the auxiliary variables (Bailey, 2022); that is, nonsampled sites are “Missing At Random” (MAR; Rubin, 1976). If the MAR assumption holds, then unbiased inference is possible. In reality, the MAR assumption is likely to be violated, because data are not available on all relevant auxiliary variables, so the best we can hope for is a reduction in bias relative to naive inferences drawn from the unadjusted sample.

The use of sample adjustments in biodiversity monitoring is variable. It is common for monitoring schemes to weight samples in such a way that the relative frequencies of habitats or geographic areas in the sample are similar to those in the population (Gregory et al., 2005; Van Swaay et al., 2002, 2008; Weiser et al., 2020). But it is also common to see samples treated as though they are representative despite clear evidence to the contrary. For example, Vellend et al. (2013) and Dornelas et al. (2014) purported to document globally representative time trends in species richness, but Gonzalez et al. (2016) showed that their samples were highly unrepresentative with respect to drivers of biodiversity change and species richness itself. (See Boyd, Powney, & Pescott, 2023 for a review of this debate and others similar to it.) We suspect that many of those who do not deal with issues of sample representativeness are not familiar with the gravity of the problem or the relevant theory and adjustment methods.

In this paper, we introduce six approaches to descriptive inference using unrepresentative nonprobability samples and demonstrate how they relate to each other (conceptually and mathematically). We apply each approach to a simple two‐part biodiversity monitoring problem. The first part is to estimate mean occupancy of the plant *C. vulgaris* across 1‐km grid squares in Britain in two time periods; the second is to estimate the difference between the two (i.e., the time trend). *Calluna vulgaris* is an attractive case study because we have good estimates of its true geographic distribution in both periods from satellite (among other sources). The approaches to the inference that we demonstrate are subsampling, quasirandomization (Elliott & Valliant, 2017), poststratification (Little, 1993), superpopulation modeling (Valliant, 2009), a “doubly robust” estimator (Chen et al., 2020), and multilevel regression and poststratification (MRP; Gelman, 2007; Gelman & Little, 1997). Each can be (MRP more loosely than the rest) interpreted as an attempt to weight the sample in such a way that it more closely resembles the population, in the hope that this results in more accurate descriptive inferences. We demonstrate the effects of each approach on the distributions of auxiliary variables in the sample, as well as on the resulting estimates of mean occupancy in each period and the time trend between the two. Applying the adjustment methods to a real‐world example reveals challenges that ecologists are likely to face, and we discuss these in detail.

## METHODS

### True distribution of *Calluna vulgaris*

**FIGURE 1:**
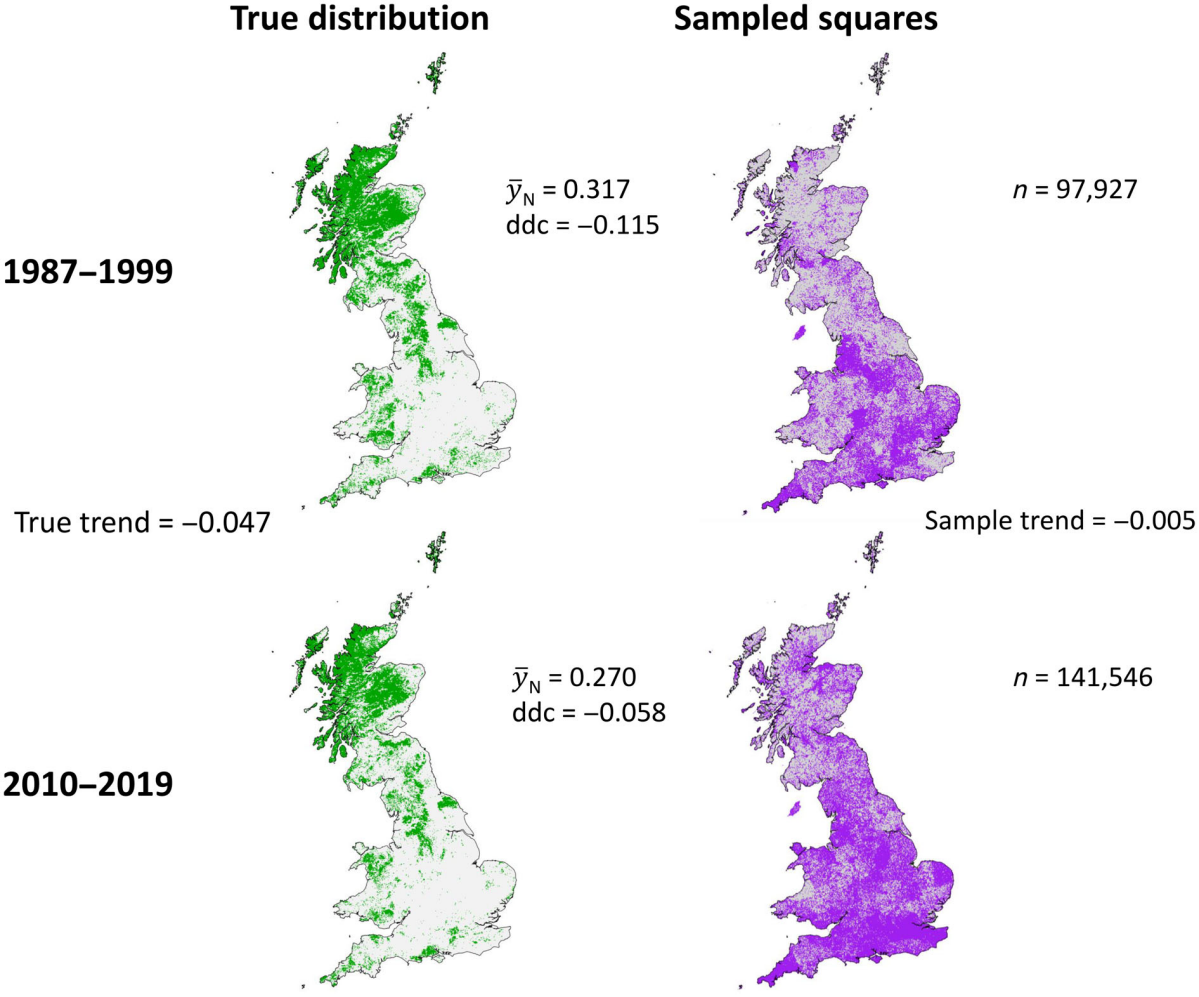
Left column: the distribution of *Calluna vulgaris* in both time periods. Green squares are occupied and gray squares are not. ${\bar{y}}_{N}$ is mean occupancy or, equivalently, the proportion of squares occupied. The ddcs are the correlations between sample inclusion (1 if the square is in the sample and 0 alternatively) and occupancy. Right column: the nonprobability 1‐km samples for each time period. Purple squares were sampled and gray squares were not. *n* is the number of squares sampled. We assumed that *C. vulgaris* was recorded in all sampled grid squares that it occupied in the relevant time period. The true trend is the difference between population means, and the sample trend is the difference between sample means (i.e., mean occupancy across purple squares).

We approximated the true distribution of the dwarf shrub vascular plant *Calluna vulgaris* (Heather) in two time periods: 1987–1999 and 2010–2019. For the first period, we used the 1990 UKCEH land cover map (Rowland et al., 2020); for the second, we used the 2018 version (Morton et al., 2022). The land cover maps are derived from satellite images, which means that they provide information for every 1‐km grid square. From these maps, we identified 1‐km grid squares (British National Grid, EPSG:27700) with >0% heather or heather grassland cover. To these, we added 1‐km squares in which *C. vulgaris* was recorded in each time period by the Botanical Society of Britain and Ireland (BSBI). The time periods considered cover the main periods of recording for two national distribution atlases, which involved a concerted effort by volunteers (citizen scientists) to document vascular plants across the United Kingdom (Preston et al., 2002; Stroh et al., 2023). Acknowledging that some 1‐km squares may have been erroneously classed as having some heather or heather grassland coverage by the land cover maps, we removed any 1‐km squares that fell within 10‐km grid squares in which *C. vulgaris* had not been recorded by the BSBI in the period 1950–2019. Given that this period included recording for three national distribution atlases (the two cited above plus Perring & Walters, 1962), we assumed that the union of all 10‐km occurrences within this period encompassed all known populations irrespective of finer scale changes. Figure 1 maps the resulting estimates of the true 1‐km distributions of *C. vulgaris* in both time periods.

### Sample data on *Calluna vulgaris* occupancy

The 1‐km samples for both time periods (sampled squares in Figure 1) encompass any vascular plant data for which the date of collection is known (i.e., the record is resolved to the day), either at the 1‐km scale or finer, collected by the BSBI for the national distribution atlases of Preston et al. (2002) and Stroh et al. (2023). Having been collected by volunteers, the data come under the banner of citizen science.

### Auxiliary data

We used two auxiliary variables for which data are available for all 1‐km grid squares in Great Britain: the proportion of each 1‐km grid square that falls within some form of protected area (including everything from SSSIs to local nature reserves; UNEP‐WCMC & IUCN, 2020) and the average elevation of each 1‐km square (Intermap, 2009). New protected areas are designated periodically, so we used the set that was designated prior to 1987 for the first time period and prior to 2010 for the second (i.e., the beginning of each period). We suspect that 1‐km squares with more protected area coverage are more likely to be visited by naturalists (Girardello et al., 2019) and, because protected areas tend to have higher quality habitat, are also more likely to be occupied by *C. vulgaris*. Similarly, elevation should affect both sample inclusion and *C. vulgaris* occupancy. Sites at higher elevations are harder to access on account of their relatively harsh terrain and remoteness, and elevation is a known predictor of *C. vulgaris* occupancy (Stroh et al., 2023).

One of the adjustment methods that we describe below, quasirandomization, requires additional covariates (we use the term “covariate” to distinguish these from the auxiliary variables as defined earlier). The method involves the estimation of sample inclusion probabilities for every 1‐km grid square in Britain. This is a matter of prediction rather than inference, because we know whether each 1‐km square was sampled (i.e., there are no missing data), so it was sensible to use a wider range of covariates. See table 1 in Boyd, Stewart, et al. (2023; version 1) for a list of the additional covariates used in this model.

### Estimating the per‐period population mean

The first step in our biodiversity monitoring problem was to estimate the mean occupancy of *C. vulgaris* in each time period. Although not usually written this way, it is helpful for what comes later to re‐express the population mean as a weighted sum:

(1) $${\bar{y}}_{N}=\frac{1}{N}\sum_{i=1}^{N}y_{i}=\sum_{i=1}^{N}\frac{y_{i}\;}{N}=\sum_{i=1}^{N}\frac{y_{i}\;w_{i}}{\sum_{N}w_{i}},$$

where y is occupancy (1 = occupied and 0 = unoccupied), N is the population size, i indexes 1‐km grid squares and $w_{i}=1/N$ (N is the same in both time periods). The denominator in the rightmost expression might seem unnecessary, because it equals 1. We have retained it to illustrate the similarity between this expression and the sample‐based estimators below, which have a similar form but whose sampling weights w do not necessarily sum to one. (We use the term “estimator” to describe a rule for estimating some quantity from a sample; here, that quantity is the population mean.) For notational simplicity, we do not index the time period, and the reader should remember that ${\bar{y}}_{N}$ is time‐period specific. In practice, y is not known for all i in the population, so sample‐based estimators of ${\bar{y}}_{N}$ are needed.

### The design‐based estimator

The design‐based estimator of the population mean, which is applicable only when a probability sample of some sort is available (Lohr, 2022), has a similar form to Equation (1):

(2) $${\bar{y}}_{\mathrm{db}}=\sum_{i=1}^{n}\frac{y_{i}\;w_{i}}{\sum_{n}w_{i}}.$$

The differences are that the sums are over the sample size n rather than N and that the weights $w_{i}$ are not necessarily constant. Rather, the weight for unit i, $w_{i}$, is equal to the reciprocal of the probability that it was included in the sample $=1/p_{i}$.

Sample inclusion probabilities are, by definition, not known for nonprobability samples, so alternative estimators are required. We present six such estimators below, three of which—quasirandomization, poststratification, and superpopulation modeling—are explicit attempts to come up with a set of weights $w_{i}$ that produce a reasonable estimate of ${\bar{y}}_{N}$ using Equation (2). The other three—a “doubly robust” estimator, subsampling, and MRP—are not, but they are conceptually similar.

### Estimators for nonprobability samples

The following estimators have been used in survey sampling to estimate population means from nonprobability samples. More details on each can be found in Valliant et al. (2018), Lumley (2010), and Lohr (2022). See Boyd (2023) for an R Markdown document containing the code to implement each of the adjustment methods.

#### Naive sample mean

When sample inclusion probabilities are unavailable, a simple option is to assume that $w_{i}=1/n$ for all i. In this case, Equation (2) gives the (naive) sample mean. As the weights are constant, the sample mean does not adjust for differences in y between the sampled and nonsampled population units. It is nevertheless widely used in biodiversity monitoring.

#### Quasirandomization

An alternative approach is to imagine that the nonprobability sample was selected probabilistically and to estimate the implied inclusion probabilities. Any binary model and covariates can be used. Once inclusion probabilities $p_{i}$ have been estimated, the weights $w_{i}=1/p_{i}$ (as in the design‐based estimator). In our example, we used random forests and several covariates (including the auxiliaries) to estimate pseudoinclusion probabilities. More complex approaches are possible and have been used to map species distributions (Johnston et al., 2020).

#### Poststratification

Another approach to estimating sampling weights is poststratification. Poststratification requires categorical auxiliary data, so continuous variables must be discretized prior to analysis (Valliant, 2020). The auxiliary variables are crossed (think contingency tables) to create poststrata. Each poststratum j has a sample size $n_{j}$ and population size $N_{j}$. The sampling weight $w_{i}$ for population unit i in poststratum j is given by $N_{j}/n_{j}$.

In our example, we split elevation into 10 categories using its deciles (i.e., cut points at the 10th and 20th percentiles, etc.). This did not make sense for the variable denoting the proportion of each grid square that falls within a protected area, because most squares took the value one or zero. We split this variable into two categories, 0 and >0; that is whether or not there is some protected land in the grid square. Discretization gave 10×2=20 poststrata.

**FIGURE 2:**
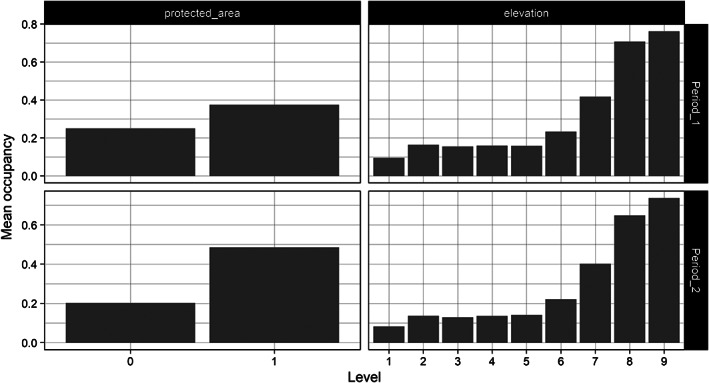
Mean occupancy of *Calluna vulgaris* for each level of the auxiliary variables in each time period. The auxiliary variables were originally on a continuous scale, but we discretized them to enable poststratification. See the main text for details.

It is sensible to discretize the auxiliary variables in such a way that the variable of interest varies among categories. Otherwise, the adjustment from poststratifying will be minor (or unnecessary)! Figure 2 shows that the mean occupancy of *C. vulgaris* in the samples differs appreciably among levels of the auxiliary variables.

#### Superpopulation modeling

Superpopulation modeling is conceptually different from the adjustment methods described above. The premise is that some model exists that describes the variable of interest in the population. If this model can be recovered from the sampled outcome variable y and the auxiliary data, it can be used to predict the variable of interest for nonsampled units. Given a prediction for each nonsampled i, it is then simple to estimate the population mean.

A general (i.e., multiple) linear regression model of y has the form

(3) $$E_{M}\left(y_{i}\right)=x_{i}^{T}\;\beta ,$$

where the subscript M indicates that the expectation (mean) is with respect to the model, $x_{i}$ is the vector of auxiliary variables for unit i, the superscript T indicates that the vector $x_{i}$ has been transposed (to a row vector) and β is a column vector of parameters. A prediction of y for unit i is

(4) $${\hat{y}}_{i}=x_{i}^{T}\;\hat{\beta }.$$

The accent on β indicates that it is an estimate (the least squares estimate in this case). If $\bar{s}$ is the set of nonsampled population units, the superpopulation model prediction of the population mean is

(5) $${\bar{y}}_{\mathrm{sp}}=\frac{\sum_{i\in s}y+\sum_{i\in \bar{s}}\hat{y}}{N}.$$

That is, it is the sum of the known outcome values in the sample and those predicted by the model for the remainder of the population divided by the population size.

A feature of ${\bar{y}}_{\mathrm{sp}}$ is that it can be expressed in the same form as the design‐based estimator in Equation (2), with the weights $w_{i}$ being a function of the auxiliary variables in sampled and nonsampled population units (Elliott & Valliant, 2017). (Code to verify this numerically is available at https://github.com/robboyd/selectionBiasEffects/tree/master/R.) As in the other adjustment models, then, the superpopulation estimator is an approach to estimating the sampling weights $w_{i}$.

Linear regression might seem like an unusual choice of model for a binary outcome (occupancy), but we felt that it was the best option here. One reason is that the implied model is actually linear for an estimator in the form of Equation (2) (Valliant, 2020). Most important, however, is that the use of a linear model enables the estimation of sampling weights (Valliant et al., 2018; supplementary material 1 in Boyd, 2023). This is helpful, because those weights can be used to show the effects of superpopulation modeling on the distributions of the auxiliary variables in the sample (see *Evaluating the effects of the adjustments* below). Alternative models can be used when weights are not required (e.g., Wu & Sitter, 2001). In our example superpopulation model, we used the auxiliary variables as predictors.

#### Doubly robust estimator

The doubly robust estimator combines the superpopulation model and the sample inclusion model from the quasirandomization procedure in such a way that, if either is correct and the sample size is large, then the estimate of the population mean is unbiased (Valliant, 2020). It has the general form (Wu, 2022)

(6) $${\bar{y}}_{\mathrm{dr}}=\frac{1}{N}\sum_{i\in s}\frac{r_{i}}{p_{i}}+\frac{1}{N}\sum_{i=1}^{N}{\hat{y}}_{i},$$

where $r_{i}=y_{i}-{\hat{y}}_{i}$ (i.e., the residuals of superpopulation model). The second term on the right is the superpopulation model prediction of ${\bar{Y}}_{N}$. If the superpopulation model is correctly specified, then it is an unbiased estimate of ${\bar{Y}}_{N}$. However, if the superpopulation model is misspecified, then the second term needs to be corrected, which is when the first term comes in. If the quasirandomization sample inclusion model is correctly specified, the first term corrects the second by adding the residuals of the superpopulation model divided by the (correctly) estimated pseudoinclusion probabilities. This is sufficient to produce an unbiased estimate of ${\bar{Y}}_{N}$, even when the superpopulation model is wrong. When the superpopulation model is correct, the first term is 0, because *r*_i_ = 0. When neither model is correct, ${\bar{y}}_{\mathrm{dr}}$ is a biased estimator of ${\bar{Y}}_{N}$. See Chen et al. (2020), who combined probability and nonprobability samples, for a similar approach.

#### Subsampling

Perhaps more familiar to ecologists than the above approaches is subsampling (Beck et al., 2014; Steen et al., 2020). The idea is to create a representative “miniature” of the population out of the sample (Meng, 2022) and to calculate the quantity of interest (mean occupancy) from this subsample. Subsampling trades sample size for representativeness.

Our approach was to draw stratified random samples of size N/10=22,958 with replacement from the original samples. We used the same strata as described above (see *Poststratification*). The decision to set n=N/10 was somewhat arbitrary, but changing the subsample size makes little difference to the point estimates of the population means (although they become more precise with increasing subsample size; supplementary material 1 in Boyd, 2023). The subsample mean is the estimator of the population mean.

#### Multilevel regression and poststratification

MRP is an extension of poststratification and a variation of superpopulation modeling (Gelman, 2007; Gelman & Little, 1997; Valliant et al., 2018). A hierarchical model is used to estimate mean occupancy in each poststratum. The advantage of using a hierarchical model is that cells with few or no data borrow information from cells with more data (i.e., partial pooling or shrinkage is exploited). The population mean is the weighted mean of the stratum means, when the weights are equal to the proportion of the population in each stratum.

Our hierarchical model is a binomial generalized linear model (GLM) with a logit link function, a fixed intercept and random intercepts for the auxiliary variables and their interaction (see https://mc-stan.org/rstanarm/articles/mrp.html for a similar formulation). We fitted the model in a Bayesian framework using five Markov Chain Monte Carlo (MCMC) chains, each with 1000 iterations. This was sufficient to achieve convergence on all parameters in both time periods.

#### Confidence intervals

We present 95% confidence/credible intervals for all estimates of mean occupancy (credible intervals for MRP, which we implemented in a Bayesian framework). For most methods—superpopulation modeling, quasirandomization, subsampling and the doubly robust estimator—we constructed bootstrap confidence intervals. Resampling the original data with replacement, we created 1000 bootstrap samples, from which we obtained a distribution of estimates from each method and calculated percentile intervals. For MRP, we extracted credible intervals from the posterior distributions of mean occupancy. We used the confidence intervals provided by the survey package (Lumley, 2010) for the poststratified and naive (i.e., unadjusted) estimates.

### Estimating the trend in mean occupancy

Having estimated mean occupancy in each time period, the next step was to estimate the difference between the two $={\bar{y}}_{2}-{\bar{y}}_{1}$ (i.e., the trend). We constructed a confidence interval for the trend estimated using each method in one of two ways depending on whether the method produced one estimate or a distribution. The methods that produced a distribution of ${\bar{y}}_{2}-{\bar{y}}_{1}$ include those that we bootstrapped and MRP, which we fitted in a Bayesian framework (meaning we have a posterior distribution). For these methods, we extracted percentile confidence intervals (95%) from the distributions of estimated trends. For the others, poststratification and the naive estimator (the sample mean), we used the normal approximation of the 95% confidence interval, given by ±1.96× the standard errors, where the standard errors are $\sqrt{\mathrm{var}\left({\bar{y}}_{2}\right)+\mathrm{var}\left({\bar{y}}_{1}\right)}$ (Gelman, 2007).

### Evaluating the effects of the adjustments

We used relative frequency plots (cf. Makela et al., 2014) to assess whether the adjustments brought the distributions of the auxiliary variables in the samples closer to their distributions in the population. The first step was to split each auxiliary variable into 50 bins of equal width spanning its range. The relative frequency of grid squares (the ′*i*′s) in each bin k is $N_{i,k}/N$, where $N_{i,k}$ is the number of grid squares in each bin k in the population and N is the population size (we use k to index the bins to distinguish them from the strata described earlier). Similarly, the relative frequency of sampled grid squares in each k is $n_{i,k}/n$, where $n_{i,k}$ is the number of sampled grid squares in bin k and n is the total sample size. In the adjusted samples, the equivalent relative frequency is $\frac{\sum_{i\in k}w_{i}}{\sum_{N}w_{i}}$ (slightly different for subsampling; see below). We compared the original and adjusted samples' deviations from the population using the mean absolute error (MAE) of the relative frequencies across all k. If the MAE from the adjusted sample is smaller than the original sample, then the adjustment brought the distribution of the auxiliary variable closer to its population distribution.

We were not able to produce adjusted relative frequency plots based on the doubly robust estimator or MRP. The problem was that we could not estimate reasonable sampling weights from either method, which are needed to adjust the relative frequencies of the auxiliaries. While it has been shown how to derive unit‐level sampling weights when the MRP multilevel model is linear (Gelman, 2007), no formula has yet been derived for the case of the binomial GLM (Valliant et al., 2018). As for the doubly robust estimator, Valliant (2020) showed how to derive “model‐assisted” weights. Unfortunately, in our case, many of the model‐assisted weights were very large and negative. The extreme weights appear to be caused by the pattern of residuals from the superpopulation model (recalling that we used a linear regression despite the fact that occupancy is binary), but it is beyond the scope of this paper to definitively diagnose the problem. There is no obvious way to derive weights from the subsampling estimator either. However, for this estimator, the adjusted relative frequencies of the auxiliaries are simply their distributions in the subsamples, so they are simple to obtain.

Assessing whether the estimates of mean occupancy in each period and the trend were improved by each adjustment method was simpler. We measured the difference between the point estimates of mean occupancy and the truth using the absolute error = $\left|{\bar{y}}_{N}-{\bar{y}}_{\mathrm{est}}\right|$, where ${\bar{y}}_{\mathrm{est}}$ is the estimate. For the trends, whose signs are of interest, we simply used the differences between the estimates and the truth. We also assessed whether the confidence/credible intervals produced by each method covered the true means and trend. We did not consider the power to detect the trend—that is, whether the methods' uncertainty intervals span zero at some percentile—because many biodiversity applications are descriptive inferential rather than decision theoretic.

## RESULTS

### Per‐period sample representativeness and estimated mean occupancy

**FIGURE 3:**
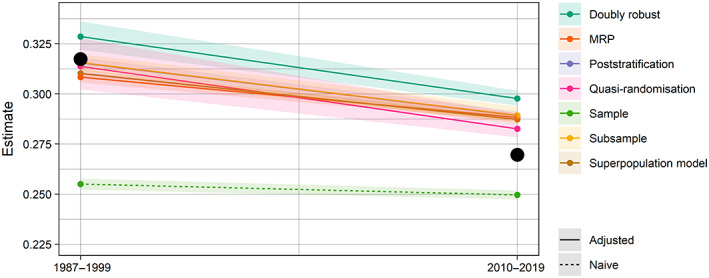
Naive (i.e., unadjusted) and adjusted sample‐based estimates of mean occupancy in each time period. The shaded regions are 95% confidence/credible intervals (see the main text for information on we constructed these for each method). MRP, multilevel regression and poststratification.

The samples are large but somewhat unrepresentative (Figure 1). 43% of grid squares were sampled in period one, and the ddc is −0.115; in period two, 62% of grid squares were sampled and the ddc is −0.057. A consequence of these ddcs is that the naive sample means underestimate the population means, especially in period one where the magnitude of the ddc is greater (Figure 3).

With the exception of the doubly robust estimate in period two, the estimates of mean occupancy from all adjustment methods in both time periods had lower absolute errors than the naive sample mean (Figure 3; MAEs are provided in supplementary material 2 in Boyd, 2023). The confidence intervals for the poststratified, subsample and quasirandomization estimates covered the true population mean in period one. In period two, no method's confidence/credible interval covered the population mean.

### Estimated trend in mean occupancy

**FIGURE 4:**
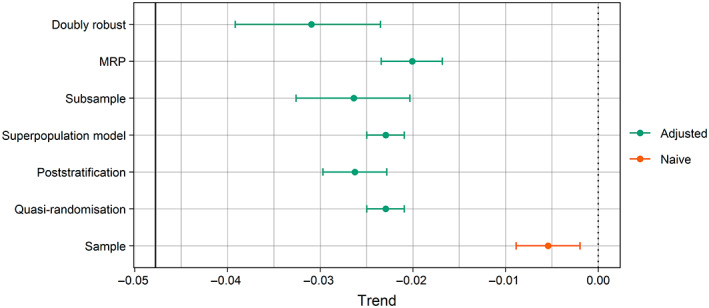
Trends in mean occupancy between periods one and two produced by the estimator from each adjustment method, in addition to the naive sample estimate. Error bars delimit 95% confidence/credible intervals. The solid vertical black line denotes the true population trend (−0.047).

Estimates of the trend in mean occupancy from all adjustment methods were more accurate than the difference in sample means (i.e., the naive estimate; Figure 4). However, no method's point estimate came close to the true trend of −0.047, and their confidence/credible intervals did not cover it.

### Distributions of auxiliary variables

**FIGURE 5:**
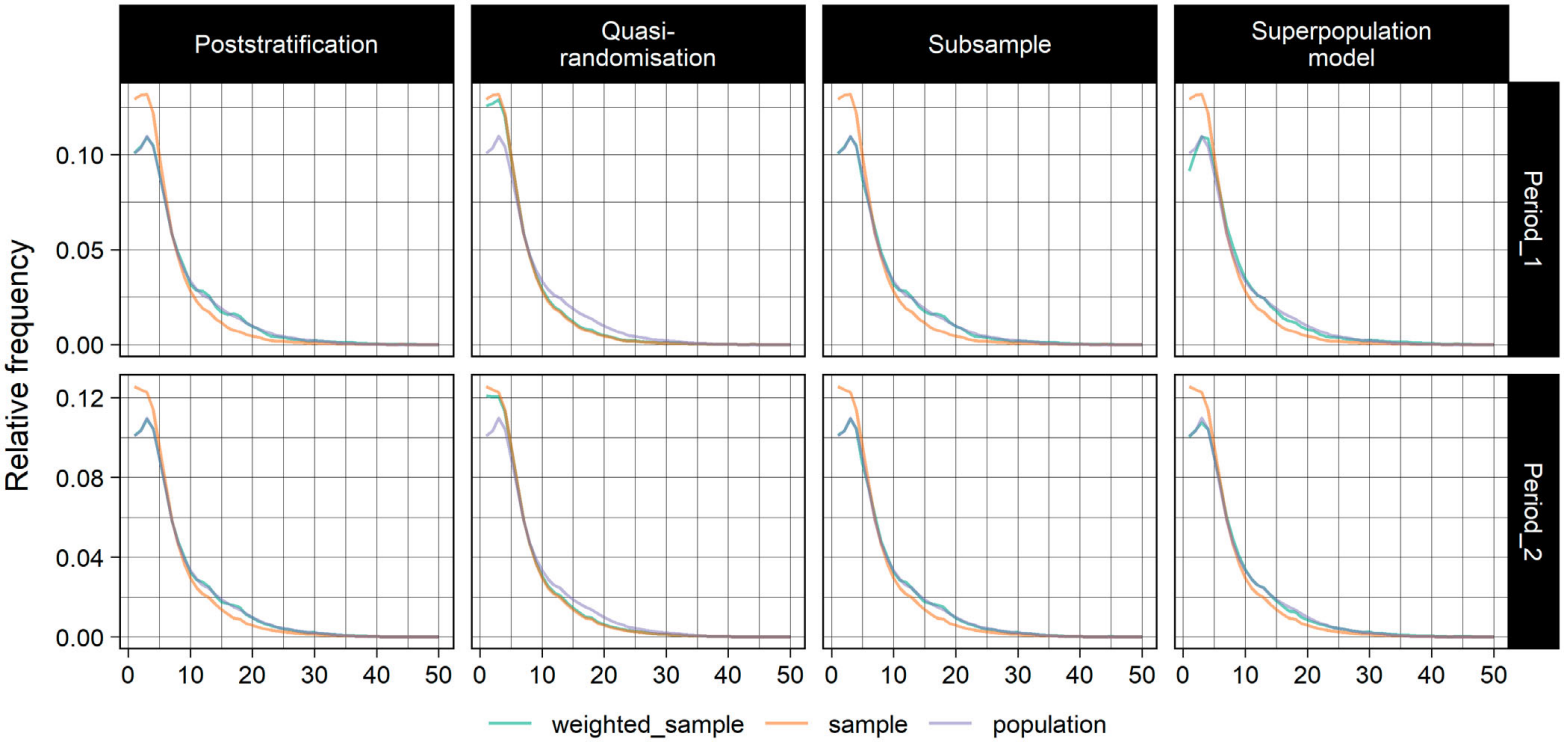
Sample, population and weighted sample distributions of the auxiliary variable road length in periods one and two.

As measured using MAEs, the adjustment methods were generally very good at bringing the distributions of the auxiliaries in the samples closer to those in the population. Figure 5 shows the sample and population distributions of elevation, but the MAEs for this and the proportion of each grid square that falls within a protected area can be found in supplementary material 2 of Boyd (2023).

## DISCUSSION

We applied six approaches to descriptive inference from nonprobability samples to a simple biodiversity monitoring problem: the estimation of mean occupancy of the plant *C. vulgaris* in two time periods and the trend between the two. The methods generally worked well in the sense that they brought the distributions of auxiliary variables in the samples closer to their distributions in the population (all 1‐km grid squares in Britain). Successful redistribution of the auxiliaries translated into improvements in the estimates of mean occupancy in both time periods and the trend between the two. Importantly, however, no method was completely unbiased, and their uncertainty intervals did not cover the true values of occupancy in the second period or the trend. An abatement rather than an elimination of bias is probably the best outcome that can be expected, because most adjustment methods rest on the untenable assumption that nonsampled locations are MAR; that is, the variable of interest is completely independent of sample inclusion given the auxiliary variables.

Unlike most practical situations, we were able to test the MAR assumption, because we know the true distribution of *C. vulgaris* in Britain. In the first time period, the partial correlation between sample inclusion and occupancy, conditional on elevation and protected area coverage, is −0.018; in period two it is 0.035 (supplementary material 1 in Boyd, 2023). These “adjusted” ddcs are lower in magnitude than the original ddcs, −0.115 and −0.058, which means that accounting for elevation and protected area coverage increased the representativeness of the samples (recalling that a smaller ddc means a more representative sample). That is not to say that the samples became fully representative, which would be the case in expectation of an MAR scenario. The usual yardstick for a representative sample is the simple random sample, whose ddc is of the order $N^{-1/2}$ (Meng, 2018). In our example, $N^{-1/2}=2.2^{-6}$, which is several orders of magnitude smaller than the “adjusted” ddcs. This goes to show that, without a truly minuscule ddc, which would only be induced (in expectation) when the MAR assumption holds or under random sampling, sample means as estimators of population means will be appreciably biased (especially where N is large).

It might seem wise to include as many potential auxiliaries as possible to reduce the chance of missing a genuine one. For example, Collins et al. (2001) advocated for including all variables exceeding some prescribed correlation with sample inclusion and the variable of interest. This strategy can be a dangerous one, however. Thoemmes and Rose (2014) showed that including correlates of sample inclusion and the variable of interest, rather than theoretically justifiable causes, can increase bias in estimates of population means (also see Thoemmes & Mohan, 2015). Indeed, in a previous version of this manuscript (Boyd, Stewart, et al., 2023), we took a more inclusive approach to the selection of auxiliary variables, and our estimates of *C. vulgaris* occupancy in period two were generally more biased than the naive estimate from the unadjusted sample.

Identifying appropriate auxiliary variables is likely to be the most challenging part of adjusting samples in biodiversity monitoring. In many situations, the causes of the variable of interest and sample inclusion are not known. Taxon and dataset experts might be able to identify potential auxiliary variables, but it is unlikely that they can identify them all (which would be needed to satisfy the MAR assumption). The experts might also erroneously identify auxiliary variables that are not suitable, in which case adjusting for those variables might do more harm than good (Thoemmes & Rose, 2014). Even if experts were able to correctly identify all relevant auxiliaries, those variables might not be reflected in available data. Transparency regarding the availability and choice of auxiliary variables should be an important component of reporting for all biodiversity monitoring.

Acknowledging that variables of interest in biodiversity monitoring are likely to be dependent on sample inclusion even after controlling for the available auxiliaries, it might be worth considering adjustment methods that forgo the MAR assumption. For example, Tchetgen Tchetgen and Wirth (2017) showed that it is possible to recover a true population regression model (and therefore the population mean) by incorporating “instrumental variables.” They define instrumental variables as those that are predictive of sample inclusion, independent of the variable of interest and independent of “selection bias” (the latter defined as the mean of the variable of interest in the sample minus the mean of the variable of interest in nonsampled population units). We screened three additional variables—the proportion of each grid square that is accessible to the public, the density of postcodes in each grid square and its nearest neighbors, and the length of major roads in each grid square and its nearest neighbors—to see if they satisfied these three assumptions, but none did (supplementary material 1 in Boyd, 2023). In practical situations, in which the variable of interest is not known for nonsampled population units, testing these assumptions would be challenging.

While we are confident that the availability of data on auxiliary variables was the limiting factor in our example, it is possible that improvements to the adjustment methods themselves could have improved matters. When sampling weights are not of interest, for example, it might be sensible to use a binomial GLM, rather than a general linear regression, for the superpopulation model (Wu & Sitter, 2001). The multilevel modeling component of MRP exploits partial pooling, so we could have used more finely resolved strata on the basis that estimates for sparse strata (with low sample sizes) would be shrunk toward those from strata with more data. The question is whether fine tuning the adjustment methods is likely to result in large improvements in accuracy. As Mercer et al., (2018), writing in the context of adjusting survey samples, put it “[t]he right variables make a big difference for accuracy. Complex statistical methods, not so much.” The fact that most adjustment methods performed similarly in our example is further evidence that the choice of auxiliary variables matters more than the specifics of the adjustment method.

Given that the methods performed similarly in terms of accuracy, it would be sensible to consider those that are quickest to run. As we implemented it, MRP took by far the longest to run of all the methods: about 10 hours per time period on a computer cluster. Bootstrapping to estimate confidence intervals meant that other methods, too, were quite expensive to run. This was particularly true for the quasirandomization and doubly robust procedures, both of which involved repeatedly fitting the sample inclusion model, itself a time‐consuming process. The remainder of the methods—superpopulation modeling, subsampling, and poststratification—took a negligible amount of time to run.

Although we have only considered one species and dataset, previous studies (in other disciplines) have shed light on the factors that affect the accuracy of inference from nonprobability samples more generally. Omitting genuine auxiliary variables in the adjustment process is more problematic when those variables explain larger proportions of the variance in the variable of interest and sample inclusion (Collins et al., 2001). Equally, the inclusion of certain variables that are not appropriate auxiliaries becomes more problematic when they explain larger proportions of the variance in the variable of interest and sample inclusion (Thoemmes & Rose, 2014). In practice, we do not know the strengths of the effects of potential auxiliaries on the variable of interest and sample inclusion, or whether they have effects at all, but it is clear that the selection of auxiliary variables will be a critical component of adjusting samples in biodiversity monitoring.

Given the importance of selecting appropriate auxiliary variables, we propose the following general strategy for analysts intending to draw inferences about biodiversity change from geographically unrepresentative nonprobability samples. The first step should be to consult taxon and dataset experts, who might be able to identify relevant auxiliary variables. When possible, consulting multiple experts to capture their uncertainty about what affects sample inclusion and the variable of interest would be desirable. If data are available on these variables, then their distributions in the sample and population should be compared with assess whether the data are representative with respect to that variable. Several tools are available to perform such comparisons (Boyd et al., 2021; Ruete, 2015). The next step should be to adjust the sample based on the relevant auxiliaries and to draw inferences from the adjusted samples. Like others (e.g., Mercer et al., 2018), we found that it is of little consequence which adjustment method is used, so it is sensible to pick one that is quick to run. Rather than assuming that the adjustment worked perfectly, it is important to acknowledge and report the potential for residual bias. As we have shown, traditional uncertainty intervals are not guaranteed (or even likely) to cover the true population parameters of interest unless all relevant auxiliaries are known and reflected in available data (Meng, 2018). When there is doubt about the relevant auxiliary variables, a safer strategy is to assess the risk of bias qualitatively and to ensure it is reflected in the way that findings are reported (Boyd et al., 2022; Meineke & Daru, 2021; Pescott et al., 2022).

## CONFLICT OF INTEREST STATEMENT

The authors declare no conflicts of interest.

## Data Availability

The data and an R Markdown document containing all code to reproduce our analysis are available on Zenodo in Boyd (2023) at https://doi.org/10.5281/zenodo.10029669.

## References

[ecy4214-bib-0001] Bailey, M. A. 2022. “Comments on ‘Statistical Inference with Non‐probability Survey Samples’.” Survey Methodology 48(12): 331–338.

[ecy4214-bib-0071] Beck, J., M. Böller, A. Erhardt, and W. Schwanghart . 2014. “Spatial Bias in the GBIF Database and Its Effect on Modeling Species' Geographic Distributions.” Ecological Informatics 19: 10–15. 10.1016/j.ecoinf.2013.11.002.

[ecy4214-bib-0002] Bethlehem, J. , F. Cobben , and B. Schouten . 2008. “Indicators for the Representativeness of Survey Response.” In Statistics Canada's International Symposium Series: Proceedings 11. Statistics Canada. https://www150.statcan.gc.ca/n1/pub/12-001-x/2009001/article/10887-eng.pdf.

[ecy4214-bib-0003] Bowler, D. E. , N. Bhandari , L. Repke , C. Beuthner , C. T. Callaghan , D. Eichenberg , K. Henle , et al. 2022. “Decision‐Making of Citizen Scientists when Recording Species Observations.” Scientific Reports 12(1): 1–12. 10.1038/s41598-022-15218-2.35773384 PMC9245884

[ecy4214-bib-0004] Bowler, D. E. , D. E. Klaus‐ , J. Conze , F. Suhling , K. Baumann , T. Benken , A. Bönsel , et al. 2021. “Winners and Losers over 35 Years of Dragonfly and Damselfly Distributional Change in Germany.” Diversity and Distributions 27: 1353–1366. 10.1111/ddi.13274.

[ecy4214-bib-0005] Boyd, R. J. 2023. “Adjusting for Bias in Biodiversity Monitoring Data (1.0).” Zenodo, Data set. 10.5281/zenodo.10029669.

[ecy4214-bib-0006] Boyd, R. J. , G. Powney , C. Carvell , and O. L. Pescott . 2021. “occAssess: An R Package for Assessing Potential Biases in Species Occurrence Data.” Ecology and Evolution 11: 16177–16187. 10.1002/ece3.8299.34824820 PMC8601935

[ecy4214-bib-0007] Boyd, R. J. , G. D. Powney , F. Burns , A. Danet , F. Duchenne , M. J. Grainger , S. G. Jarvis , et al. 2022. “ROBITT: A Tool for Assessing the Risk‐of‐Bias in Studies of Temporal Trends in Ecology.” Methods in Ecology and Evolution 13: 1497–1507. 10.1111/2041-210X.13857.36250156 PMC9541136

[ecy4214-bib-0008] Boyd, R. J. , G. D. Powney , and O. L. Pescott . 2023. “We Need to Talk about Nonprobability Samples.” Trends in Ecology & Evolution 38(6): 521–531. 10.1016/j.tree.2023.01.001. 36775795

[ecy4214-bib-0009] Boyd, R. J. , G. B. Stewart , and O. L. Pescott . 2023. “Descriptive Inference Using Large, Unrepresentative Nonprobability Samples: An Introduction for Ecologists [Version 1].” Ecoevorxiv. 10.32942/X2359P. PMC10929663 38088061

[ecy4214-bib-0010] Chen, Y. , P. Li , and C. Wu . 2020. “Doubly Robust Inference with Nonprobability Survey Samples.” Journal of the American Statistical Association 115(532): 2011–2021. 10.1080/01621459.2019.1677241.

[ecy4214-bib-0011] Collins, L. M. , J. Schafer , and C. Kam . 2001. “A Comparison of Restrictive Strategies in Modern Missing Data Procedures.” Psychological Methods 6: 330–351. 10.1037/1082-989X.6.4.330. 11778676

[ecy4214-bib-0012] Dornelas, M. , N. J. Gotelli , B. McGill , H. Shimadzu , F. Moyes , C. Sievers , and A. E. Magurran . 2014. “Assemblage Time Series Reveal Biodiversity Change but Not Systematic Loss.” Science 344(6181): 296–299. 10.1126/science.1248484. 24744374

[ecy4214-bib-0013] Elliott, M. R. , and R. Valliant . 2017. “Inference for Nonprobability Samples.” Statistical Science 32(2): 249–264. 10.1214/16-STS598.

[ecy4214-bib-0014] Forister, M. L. , S. H. Black , C. S. Elphick , E. M. Grames , C. A. Halsch , C. B. Schultz , and D. L. Wagner . 2023. “Missing the Bigger Picture: Why Insect Monitoring Programs Are Limited in their Ability to Document the Effects of Habitat Loss.” Conservation Letters 16: e12951. 10.1111/conl.12951.

[ecy4214-bib-0015] Geldmann, J. , J. Heilmann‐Clausen , T. E. Holm , I. Levinsky , B. Markussen , K. Olsen , C. Rahbek , and A. P. Tøttrup . 2016. “What Determines Spatial Bias in Citizen Science? Exploring Four Recording Schemes with Different Proficiency Requirements.” Diversity and Distributions 22(11): 1139–1149. 10.1111/ddi.12477.

[ecy4214-bib-0016] Gelman, A. 2007. “Struggles with Survey Weighting and Regression Modeling.” Statistical Science 22(2): 153–164. 10.1214/088342306000000691.

[ecy4214-bib-0017] Gelman, A. , and T. Little . 1997. “Poststratification into Many Categories Using Hierarchical Regression.” Survey Methodology 23(2): 127–335.

[ecy4214-bib-0018] Girardello, M. , A. Chapman , R. Dennis , L. Kaila , P. A. V. Borges , and A. Santangeli . 2019. “Gaps in Butterfly Inventory Data: A Global Analysis.” Biological Conservation 236: 289–295. 10.1016/j.biocon.2019.05.053.

[ecy4214-bib-0019] Gonzalez, A. , B. J. Cardinale , G. R. H. Allington , J. Byrnes , K. A. Endsley , D. G. Brown , D. U. Hooper , F. Isbell , M. I. O'Connor , and M. Loreau . 2016. “Estimating Local Biodiversity Change: A Critique of Papers Claiming no Net Loss of Local Diversity.” Ecology 97(8): 1949–1960. 10.1890/15-1759.1. 27859190

[ecy4214-bib-0020] Gregory, R. D. , A. Van Strien , P. Vorisek , A. W. G. Meyling , D. G. Noble , R. P. B. Foppen , and D. W. Gibbons . 2005. “Developing Indicators for European Birds.” Philosophical Transactions of the Royal Society B: Biological Sciences 360(1454): 269–288. 10.1098/rstb.2004.1602. PMC1569455 15814345

[ecy4214-bib-0021] Hudson, L. N. , T. Newbold , S. Contu , S. L. L. Hill , I. Lysenko , D. Palma , H. R. P. Phillips , et al. 2014. “The PREDICTS Database: A Global Database of how Local Terrestrial Biodiversity Responds to Human Impacts.” Ecology and Evolution 4: 4701–4735.25558364 10.1002/ece3.1303PMC4278822

[ecy4214-bib-0022] Hughes, A. , M. Orr , K. Ma , M. Costello , J. Waller , P. Provoost , C. Zhu , and H. Qiao . 2020. “Sampling Biases Shape our View of the Natural World.” Ecography 44: 1259–1269. 10.1111/ecog.05926.

[ecy4214-bib-0023] Intermap . 2009. “NEXTMap British Digital Terrain 50 m Resolution (DTM10) Model Data by Intermap.” NERC Earth Observation Centre. https://catalogue.ceda.ac.uk/uuid/f5d41db1170f41819497d15dd8052ad2.

[ecy4214-bib-0024] Johnston, A. , E. Matechou , and E. B. Dennis . 2022. “Outstanding Challenges and Future Directions for Biodiversity Monitoring Using Citizen Science Data.” Methods in Ecology and Evolution 14: 103–116. 10.1111/2041-210X.13834.

[ecy4214-bib-0025] Johnston, A. , N. Moran , A. Musgrove , D. Fink , and S. R. Baillie . 2020. “Estimating Species Distributions from Spatially Biased Citizen Science Data.” Ecological Modelling 422: 108927. 10.1016/j.ecolmodel.2019.108927.

[ecy4214-bib-0072] Little, R. J. A. 1993. “Post‐Stratification: A Modeler's Perspective.” Journal of the American Statistical Association 88(Sep): 1001–1012.

[ecy4214-bib-0026] Lohr, S. 2022. Sampling: Design and Analysis, 3rd ed. Boca Raton, FL: CRC Press.

[ecy4214-bib-0027] Lumley, T. 2010. Complex Surveys: A Guide to Analysis Using R, 1st ed. Hoboken, NJ: Wiley.

[ecy4214-bib-0028] Makela, S. , Y. Si , and A. Gelman . 2014. “Statistical Graphics for Survey Weights.” Revista Colombiana de Estadística 37: 285–295. 10.15446/rce.v37n2spe.47937.

[ecy4214-bib-0029] Mandeville, C. P. , E. B. Nilsen , and A. G. Finstad . 2022. “Spatial Distribution of Biodiversity Citizen Science in a Natural Area Depends on Area Accessibility and Differs from Other Recreational Area Use.” Ecological Solutions and Evidence 3(4): 1–14. 10.1002/2688-8319.12185.

[ecy4214-bib-0030] McClure, C. J. W. , and B. W. Rolek . 2023. “Pitfalls Arising from Site Selection Bias in Population Monitoring Defy Simple Heuristics.” Methods in Ecology and Evolution 14(6): 1489–1499. 10.1111/2041-210X.14120.

[ecy4214-bib-0031] Meineke, E. K. , and B. H. Daru . 2021. “Bias Assessments to Expand Research Harnessing Biological Collections.” Trends in Ecology & Evolution 36(12): 1071–1082. 10.1016/j.tree.2021.08.003. 34489117

[ecy4214-bib-0032] Meng, X.‐L. 2018. “Statistical Paradises and Paradoxes in Big Data (I): Law of Large Populations, Big Data Paradox, and the 2016 us Presidential Election.” Annals of Applied Statistics 12(2): 685–726. 10.1214/18-AOAS1161SF.

[ecy4214-bib-0033] Meng, X.‐L. 2022. “Comments on the Wu (2022) Paper by Xiao‐Li Meng 1: Miniaturizing Data Defect Correlation: A Versatile Strategy for Handling Non‐probability Samples.” Survey Methodology 48(2): 1–22.

[ecy4214-bib-0034] Mercer, A. , A. Lau , and C. Kennedy . 2018. “For Weighting Online Opt‐in Samples, What Matters Most?” In Pew Research Center 1–55. https://pewrsr.ch/3heqknn.

[ecy4214-bib-0035] Morton, R. , C. Marston , A. O'Neil , and C. Rowland . 2022. “Land Cover Map 2018 (1 km Summary Rasters, GB and N. Ireland).” NERC EDS Environmental Information Data Centre. 10.5285/9b68ee52-8a95-41eb-8ef1-8d29e2570b00.

[ecy4214-bib-0036] Nelson, G. , and S. Ellis . 2019. “The History and Impact of Digitization and Digital Data Mobilization on Biodiversity Research.” Philosophical Transactions of the Royal Society B: Biological Sciences 374(1763): 2–10. 10.1098/rstb.2017.0391. PMC6282090 30455209

[ecy4214-bib-0037] Outhwaite, C. , R. D. Gregory , R. E. Chandler , B. Collen , and N. J. B. Isaac . 2020. “Complex Long‐Term Biodiversity Change among Invertebrates, Bryophytes and Lichens.” Nature Ecology & Evolution 4: 384–392. 10.1038/s41559-020-1111-z. 32066888

[ecy4214-bib-0038] Perring, F. , and S. Walters . 1962. Atlas of the British Flora. Thomas Nelson & Sons. https://books.google.co.uk/books/about/Atlas_of_the_British_Flora.html?id=2kWJzgEACAAJ&redir_esc=y.

[ecy4214-bib-0039] Pescott, O. L. , P. A. Stroh , T. A. Humphrey , and K. J. Walker . 2022. “Simple Methods for Improving the Communication of Uncertainty in Species’ Temporal Trends.” Ecological Indicators 141: 109117. 10.1016/j.ecolind.2022.109117.

[ecy4214-bib-0040] Pescott, O. L. , K. J. Walker , F. Harris , H. New , C. M. Cheffings , N. Newton , M. Jitlal , J. Redhead , S. M. Smart , and D. B. Roy . 2019. “The Design, Launch and Assessment of a New Volunteer‐Based Plant Monitoring Scheme for the United Kingdom.” PLoS One 14(4): 1–30. 10.1371/journal.pone.0215891. PMC6485706 31026278

[ecy4214-bib-0041] Pescott, O. L. , K. J. Walker , M. J. O. Pocock , M. Jitlal , C. L. Outhwaite , C. M. Cheffings , F. Harris , and D. B. Roy . 2015. “Ecological Monitoring with Citizen Science: The Design and Implementation of Schemes for Recording Plants in Britain and Ireland.” Biological Journal of the Linnean Society 115(3): 505–521. 10.1111/bij.12581.

[ecy4214-bib-0042] Powney, G. D. , C. Carvell , M. Edwards , R. K. A. Morris , H. E. Roy , B. A. Woodcock , and N. J. B. Isaac . 2019. “Widespread Losses of Pollinating Insects in Britain.” Nature Communications 10(2019): 1–6. 10.1038/s41467-019-08974-9. PMC6435717 30914632

[ecy4214-bib-0043] Preston, C. D. , D. A. Pearman , and T. D. Dines , eds. 2002. New Atlas of the British and Irish Flora. Oxford: Oxford University Press.

[ecy4214-bib-0044] Rowland, C. , C. Marston , R. Morton , and A. O'Neil . 2020. “Land Cover Map 1990 (1 Km Dominant Target Class, GB) v2.” NERC EDS Environmental Information Data Centre. 10.5285/f5e3bd00-efd0-4dc6-a454-aa597d84764a.

[ecy4214-bib-0045] Rubin, D. B. 1976. “Inference and Missing Data.” Biometrika 63(3): 581–592. 10.1093/biomet/63.3.581.

[ecy4214-bib-0046] Ruete, A. 2015. “Displaying Bias in Sampling Effort of Data Accessed from Biodiversity Databases Using Ignorance Maps.” Biodiversity Data Journal 3(1): 1–15. 10.3897/BDJ.3.e5361. PMC4549634 26312050

[ecy4214-bib-0073] Steen, V. A. , M. W. Tingley , P. Paton , and C. Elphick . 2020. “Spatial Thinning and Class Balancing: Key Choices Lead to Variation in the Performance of Species Distribution models with Citizen Science Data.” Methods in Ecology and Evolution 12(December): 216–226. 10.1111/2041-210X.13525.

[ecy4214-bib-0047] Stroh, P. A. , K. Walker , T. A. Humphrey , O. L. Pescott , and R. Burkmar . 2023. Plant Atlas 2020: Mapping Changes in the Distribution of the British and Irish Flora. Princeton: Princeton University Press.

[ecy4214-bib-0048] Tchetgen Tchetgen, E. J. , and K. E. Wirth . 2017. “A General Instrumental Variable Framework for Regression Analysis with Outcome Missing Not at Random.” Biometrics 73(4): 1123–1131. 10.1111/biom.12670.28230909 PMC5569006

[ecy4214-bib-0049] Thoemmes, F. , and K. Mohan . 2015. “Graphical Representation of Missing Data Problems.” Structural Equation Modeling 22(4): 631–642. 10.1080/10705511.2014.937378.

[ecy4214-bib-0050] Thoemmes, F. , and N. Rose . 2014. “A Cautious Note on Auxiliary Variables that Can Increase Bias in Missing Data Problems.” Multivariate Behavioral Research 49(5): 443–459. 10.1080/00273171.2014.931799. 26732358

[ecy4214-bib-0051] UK PoMS . 2023. “The UK PoMS Annual Report 2022.” UK Centre for Ecology and Hydrology and Joint Nature Conservation Committee. https://ukpoms.org.uk/reports.

[ecy4214-bib-0052] UNEP‐WCMC, & IUCN . 2020. “Protected Planet: The World Database on Protected Areas (WDPA)/The Global Database on Protected Areas Management Effectiveness.” https://www.protectedplanet.net/en/thematic-areas/wdpa.

[ecy4214-bib-0074] Valliant, R. 2009. “Model‐Based Prediction of Finite Population Totals.” Handbook of Statistics 29(PB): 11–31. 10.1016/S0169-7161(09)00223-5.

[ecy4214-bib-0053] Valliant, R. 2020. “Comparing Alternatives for Estimation from Nonprobability Samples.” Journal of Survey Statistics and Methodology 8(2): 231–263. 10.1093/jssam/smz003.

[ecy4214-bib-0054] Valliant, R. , J. A. Dever , and F. Kreuter . 2018. Practical Tools for Designing and Weighting Survey Samples, 2nd ed. Cham: Springer. 10.1007/978-3-319-93632-1.

[ecy4214-bib-0055] van Strien, A. J. , and R. H. A. van Grunsven . 2023. “In the Past 100 Years Dragonflies Declined and Recovered by Habitat Restoration and Climate Change.” Biological Conservation 277: 109865. 10.1016/j.biocon.2022.109865.

[ecy4214-bib-0056] Van Swaay, C. A. M. , P. Nowicki , J. Settele , and A. J. Van Strien . 2008. “Butterfly Monitoring in Europe: Methods, Applications and Perspectives.” Biodiversity and Conservation 17(14): 3455–3469. 10.1007/s10531-008-9491-4.

[ecy4214-bib-0057] Van Swaay, C. A. M. , C. L. Plate , and A. J. Van Strien . 2002. “Monitoring Butterflies in The Netherlands: How to Get Unbiased Indices.” Proceedings of the Section Experimental and Applied Entomology of the Netherlands Entomological Society 13: 21–27.

[ecy4214-bib-0058] Vellend, M. , L. Baeten , I. H. Myers‐Smith , S. C. Elmendorf , R. Beauséjour , C. D. Brown , P. De Frenne , K. Verheyen , and S. Wipf . 2013. “Global Meta‐Analysis Reveals no Net Change in Local‐Scale Plant Biodiversity over Time.” Proceedings of the National Academy of Sciences of the United States of America 110(48): 19456–19459. 10.1073/pnas.1312779110.24167259 PMC3845118

[ecy4214-bib-0059] Weiser, E. L. , J. E. Diffendorfer , L. Lopez‐Hoffman , D. Semmens , and W. E. Thogmartin . 2020. “Challenges for Leveraging Citizen Science to Support Statistically Robust Monitoring Programs.” Biological Conservation 242: 108411. 10.1016/j.biocon.2020.108411.

[ecy4214-bib-0060] Wu, C. 2022. “Statistical Inference With Non‐Probability Survey Samples.” In Survey Methodology, Catalogue No. 12‐001‐X. Vol. 48, No. 2. Statistics Canada. Paper available at https://www.statcan.gc.ca/pub/12‐001‐x/2022002/article/00002‐eng.htm.

[ecy4214-bib-0061] Wu, C. , and R. R. Sitter . 2001. “A Model‐Calibration Approach to Using Complete Auxiliary Information from Survey Data.” Journal of the American Statistical Association 96(453): 185–193. 10.1198/016214501750333054.

